# The Effect of Annealing Temperatures on Selected Properties of WC/C Coatings, Deposited Using Hexacarbonyl Wolfram in an N_2_-SiH_4_ Atmosphere

**DOI:** 10.3390/ma14164658

**Published:** 2021-08-18

**Authors:** Peter Horňák, Daniel Kottfer, Karol Kyzioł, Marianna Trebuňová, Mária Kaňuchová, Lukasz Kaczmarek, Jozef Jasenák, Ján Hašuľ, Lukáš Rusinko

**Affiliations:** 1 Department of Materials Science, Faculty of Industrial Technologies, Alexander Dubček University of Trenčín, I. Krasku 491/30, 020 01 Púchov, Slovakia; hornak.peter@gmail.com; 2 Slovak Academy of Sciences, Institute of Materials Research, Watsonova 47, 040 01 Košice, Slovakia; 3 Department of Mechanical Technologies and Materials, Faculty of Special Technology, Alexander Dubček University of Trenčín, Ku Kyselke 469, 911 06 Trenčín, Slovakia; jozef.jasenak@tnuni.sk; 4 Faculty of Materials Science and Ceramics, AGH University of Science and Technology, A. Mickiewicza 30 Av., 30-059 Kraków, Poland; kyziol@agh.edu.pl; 5 Department of Biomedical Engineering and Measurement, Faculty of Mechanical Engineering, Technical University of Košice, Letná 9, 042 00 Košice, Slovakia; marianna.trebunova@tuke.sk; 6 Process Control and Geotechnology, e Institute of Mountainous Sciences and Environmental Protection, Faculty of Mining, Ecology, Technical University of Košice, Park Komenskeho 19, 043 84 Košice, Slovakia; maria.kanuchova@tuke.sk; 7 Institute of Materials Science and Engineering, Lodz University of Technology, 1/15 Stefanowskiego Str., 90-924 Łódź, Poland; lukasz.kaczmarek@p.lodz.pl; 8 Department of Technologies, Materials and Computer Aided Production, Faculty of Mechanical Engineering, Technical University of Košice, Mäsiarska 74, 040 01 Košice, Slovakia; jan.hasul@tuke.sk (J.H.); lukas.rusinko@student.tuke.sk (L.R.)

**Keywords:** WC/C coating, hexacarbonyl wolfram, plasma enhanced chemical vapor deposition, plasma, annealing, surface properties

## Abstract

In this paper, we present the results of an experimental study on WC/C coatings, deposited by using plasma-enhanced chemical vapor deposition in an N_2_-SiH_4_ atmosphere, annealed at temperatures of 200, 500 and 800 °C, in which the hexacarbonyl of W was used as a precursor. During the experiments, the topography, chemical composition, morphology, as well as selected mechanical properties, such as hardness, Young’s modulus, and coefficient of friction of the WC/C coatings were analyzed. Annealing without the protective atmosphere in the mentioned temperatures caused a decrease in hardness (up to 15 ± 2.7 GPa). In addition, the coefficient of friction value increased only to 0.37 ± 0.03.

## 1. Introduction

Tungsten carbide (WC) is often used as a thin coating, characterized by high values of hardness (42 GPa [1,2,3]), wear resistance, as well as a low coefficient of friction (COF, 0.22 [1,2]), whereas the surface hardness of unmodified steel is 5 GPa [4]. Therefore, WC is often applied for the protection of functional surfaces of machining components made of steel, and for coating high-speed steel. WC can be deposited using the physical vapor deposition (PVD) method; WC’s melting point is notably high (2870 °C) [5]. Deposition techniques, in many instances, include direct current magnetron sputtering (DCMS) [6,7,8,9,10,11,12,13] and radio frequency magnetron sputtering (RFMS) [1,14,15,16,17,18]. Reactive sputtering using W, C, or WC target is frequently carried out with additional gases, such as hydrocarbon [6], N_2_, or SiH_4_ [7,8,17]. In addition to other existing progressive PVD sputtering methods, two other methods for such WC coatings are high target utilization sputtering (HiTUS) [18], high-power impulse magnetron sputtering (HiPIMS) [7], and the chemical vapor deposition (CVD) technique [19,20].

Importantly, during the coating deposition process using CVD (also called high-temperature HT CVD) methods, the temperature of the process changes from 800 to 1200 °C, which excludes some steels and Al alloys from the deposition process.

The deposition temperature can be decreased using transitive gases, which use ionized gas (also called low-temperature and low-pressure plasma) on the coated surface. The kinetic energy of the gas particles increases and the temperature of the process can reach up to 500 °C. This process can be used in an improved CVD method, which is also called the plasma-enhanced (plasma-activated (PA), or plasma-inducted (PI)) chemical vapor deposition (PE CVD, PA CVD, or PI CVD) method. During plasmochemical processes (e.g., based on Ar and N_2_) the gas mixture is ionized and kinetic energy is transmitted to the used precursors, for example, wolfram carbonyl [21,22,23,24,25,26,27,28,29,30,31], chromium carbonyl [23,26,28,30,32,33,34,35], and molybdenum carbonyl [21,29,30,31,36], which sublime in the reactor at low temperatures.

Importantly, Garner et al. [26] described the lawfulness of W, Cr, and Mo carbonyl decompositions, while Rezuchina et al. [28] studied the relation of saturated vapor pressures and sublimation temperatures of W, Cr, and Mo carbonyls. Additionally, Chellappa and Chandra [30] described data relating to the pressure of W, Cr, and Mo carbonyls. The production and decomposition of Mo(CO)_6_ and W(CO)_6_ were presented by Usoltsev et al. [31].

The properties of WC/C coatings deposited by using PE CVD methods have been widely studied [22,23,24,25,27], as well as the properties of the Cr/C [33,34,35] and MoC/C [29] layers. To obtain a higher value of surface hardness and lower COF of WC/C thin coatings, C_2_H_2_ [23] and N_2_ [8,17,27] gases have been used during the deposition process.

In this study, based on our previous experiments [27], we focused on the microstructure, chemical composition, and selected useful properties of WC/C coatings deposited using an N_2_-SiH_4_ gas mixture and tested after annealing at various temperatures (200, 500, and 800 °C); the selected mechanical and tribological properties (i.e., hardness, Young’s modulus, and the coefficient of friction) of the deposited WC/C coatings were analyzed. The obtained results are discussed and compared with the results of other studies published in the literature on this field.

## 2. Materials and Methods

### 2.1. Sample Preparation

For the deposition process of the WC/C coatings, two types of substrates were used, monocrystal of Si (20 × 15 × 1 mm, for evaluating thickness and structure) and construction steel C45 (AISI 1045, for evaluating tribological tests). The chemical composition (wt.%) of the steel substrate is as follows: 0.42 ÷ 0.50 C, 0.40 Si, 0.50 ÷ 0.80 Mn, 0.40 Cr, 0.10 Mo, 0.4 Ni, and 0.035 P, according to the Slovak Technical Standards (STN 412050) [27]. The steel samples were cut using wire electrical discharge machining from bars with diameters of 50 and 25 mm and machined to a thickness of 3.00 ± 0.05 mm. Furthermore, the substrates were case hardened in oil after being heated to 860 °C, and subsequently annealed at 200 °C. After thermal processing, the substrates were polished using diamond pastes with granularity equal to 15, 9, and 3 µm. Finally, the samples were polished using 1 µm diamond paste to obtain surface roughness (R_a_) values of 12 nm. In the next stage, the substrates were cleaned in acetone using an ultrasonic batch for 10 min and dried in air for 5 min. Following this, the samples were inserted into a vacuum chamber and etched in Ar plasma at a pressure 2 Pa, where bias of the holder was U_b_ ≈ −5 kV, current density was 1 mA∙cm^−2^, and the time was 15 min [27]. In addition, an Ar flow of 65 cm^3^∙min^−^^1^ in the vacuum chamber was applied.

### 2.2. Coating Deposition and Annealing

The coating was deposited by adding Ar (as the working gas) in the N_2_-SiH_4_ gas mixture, where the content of SiH_4_ (silane) was 1.5%. Table 1 summarizes the technological parameters of the deposition process (which lasted for 2 h) and the selected mechanical properties of the obtained coating before annealing.

**Figure 1:**
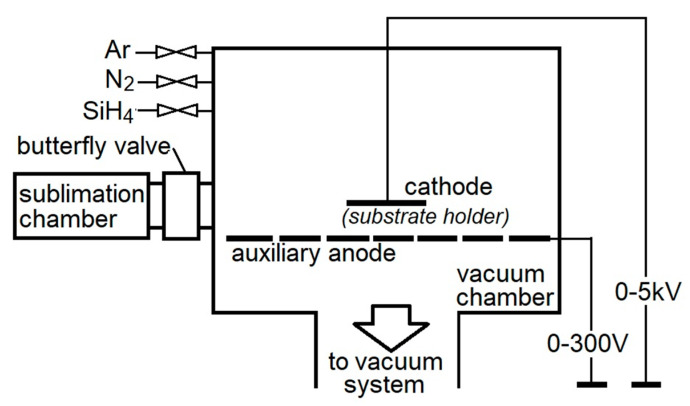
Scheme of the PE CVD equipment used during coating deposition, ZIP 12 type with sublimation chamber.

The WC/C coatings were deposited in a ZIP 12 apparatus (NTC New Technology Centre, Košice, SK, Slovakia) with a sublimation chamber (Figure 1) using the PE CVD method by applying direct-current electric voltage [27].

The substrates were placed on the cathode (substrate holder), where the maximum temperature of the deposition was controlled using Kapton tape (made from polyimide with a silicone sticking surface on one side). During the experiments, the crucible with the precursor was put into the sublimation chamber and closed using a butterfly valve between the sublimation chamber and the vacuum chamber. The cleaning procedure was performed under a pressure of 10^−3^ Pa using Ar (purity of 99.999%) as the working gas. In the next stage, during the WC/C coating deposition, the W(CO)_6_ (hexacarbonyl of W) was used.

According to [29], during the coating deposition process, the W(CO)_6_ decomposes into W + 6 CO, and then carbon monoxide is decomposed according to the Boudouard reaction 2CO →C + CO_2_. These reactions occur simultaneously in the plasma and on the substrate.

Carbon created in this manner reacts with wolfram and, as a result, creates wolfram carbide (tungsten carbide, WC). Then, tungsten carbide is deposited onto the substrate with the influence of bias on the substrate (cathode).

Annealing of the WC/C coated substrates was carried out in an electric furnace at temperatures of 200, 500 and 800 °C, without a protective atmosphere. The duration of the annealing at the mentioned temperatures was 1 h. After heating, the substrates were left to cool down.

### 2.3. Nanohardness and Young’s Modulus

After the deposition on steel substrates, selected mechanical properties of the WC/C coatings (specifically, hardness (H_IT_), and indentation (E_IT_) modulus) were measured using the instrumental indentation method (Nanoindenter NHT, CSM Instruments, Basel, Switzerland). The measurements were provided using sinus mode with an amplitude equal to 1 mN, applied load of 20–60 mN, and frequency of 15 Hz. The values of indentation hardness and indentation modulus were calculated as an average of maximal values of indentation curves.

### 2.4. Coefficient of Friction

The COF measurements of the WC/C coatings prepared on the metal substrates were performed using a ball-on-disc method on a HTT tribometer (CSM Instruments, Basel, Switzerland). The following parameters were used for the tests: unencumbered strength, 0.5 N; temperature, 21 °C; without a protective atmosphere, in the air; the counterpart (a steel ball) was made using 100 Cr6 steel with a diameter of 6 mm, a velocity of the balls’ movements of 10 cm/s, and path length of 50 m. The coefficient of friction was continuously measured in the course of every test as a function of time (in some cases as a function of the number of rotations) of the substrate and path length.

### 2.5. SEM, AFM, and XPS Analyses

The surface morphology of the evaluated coatings and their microstructures were observed using an electron microscope JEOL JSM 7000F (Tokyo, Japan).

The topography of the coated surface was measured by using an atomic force microscope (AFM) Dimension Icon, Veeco (Plainview, NY, USA).

The thicknesses of the coatings were evaluated using an electron microscope JEOL JSM 7000F by observing a transversal fracture of the WC/C coatings deposited on the Si substrates.

The chemical composition and selected atomic groups of the tested structures were observed using X-ray photoelectron spectroscopy (XPS). The XPS was performed on an instrument SPECS (SPECS GmbH, Berlin, Germany) equipped with a PHOIBOS 100 SCD and a non-monochromatic X-ray source. The survey surface spectra of samples were measured at 70 eV transition energy and core spectra at 30 eV, at room temperature. The spectra were all obtained at a basic pressure of 1 × 10^−8^ mbar with MgKα excitation at 10 kV (200 W). and the data analyses were performed using SpecsLab2 CasaXPS software (Casa Software Ltd., Teignmouth, UK). A Shirley and Tougaard type of baseline was used for all peak fits.

## 3. Results and Discussion

### 3.1. Thickness, Morphology, and Chemical Composition

**Figure 2:**
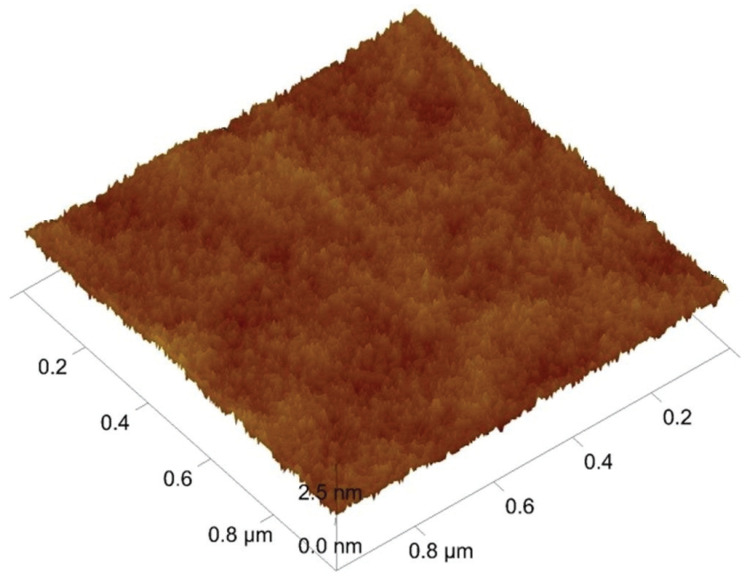
The AFM image (3D) of the unannealed WC/C coating.

**Figure 3:**
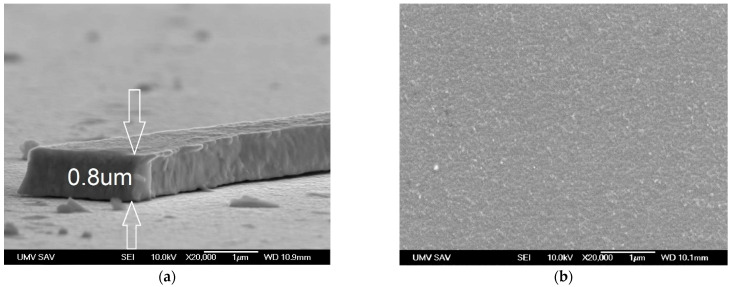

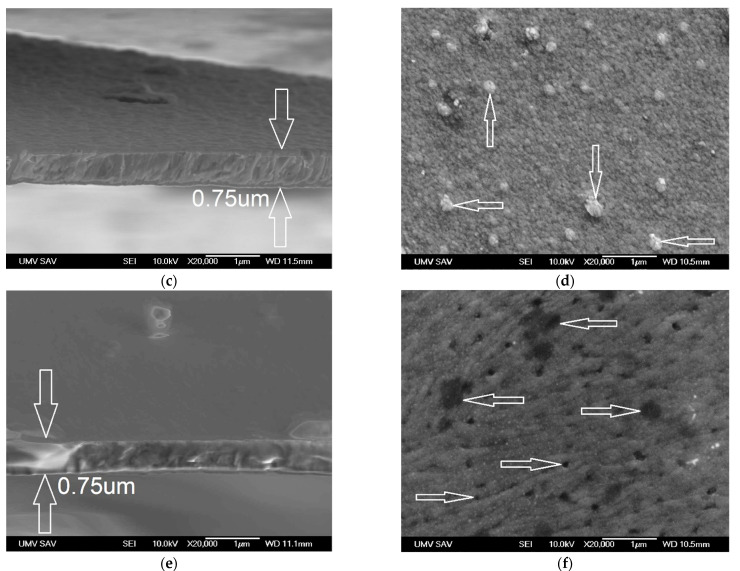
SEM images of the cross-sectional (left) and surface morphology (right) of WC/C coatings: (**a**,**d**) unannealed; (**b**,**e**) after annealing at 500 °C; (**c**,**f**) after annealing at 800 °C.

Figure 2 and Figure 3a, d show the AFM and SEM images of deposited WC/C coating characterized by a nanocolumnar structure. The thickness of the deposited coating was 0.75 ± 0.05 µm (Figure 3a). After annealing at a temperature of 500 °C, the beginning of the coating degradation process can be observed, Figure 3b,e. The diameter of grains increased and, in places marked with arrows, column structures and the presence of globulites with a size up to 200 nm are visible, which point toward signs of coating degradation caused by swelling. Swelling is caused by the reaction between C within the coating and oxygen from the atmosphere in an environment with higher temperatures. Next, CO_2_ is created, which swells within the coating. The outcome is a degraded coating in the whole cross-section, such as that shown by Lofaj F. and Kaganowskyy Yu.S. [37]. On the other hand, it can appear within the whole cross-section, but in small volumes (Figure 3c). As a result of annealing at 800 °C (Figure 3f) and swelling, empty spaces with varying sizes ranging from 0.1 to 0.5 µm (see arrows) are created. Despite this, the degradation of the WC/C coating deposited with the N_2_-SiH_4_-Ar gas mixture is significantly lower as compared with other study results [8,17], where massive degradation of WC/C coatings as a result of swelling alongside the whole cross-section have occurred, such as that shown by Lofaj F. and Kaganowskyy Yu.S. [37]. This indicates that the significant increase in the refractoriness of the WC/C coatings at the mentioned temperatures is the result of adding 1.5% SiH_4_ (silane) to N_2_ during the PE CVD process as compared with the WC/C coatings deposited by applying the N_2_-SiH_4_ gas mixture using DCMS [8] and RFMS [17] methods. The thickness of the coating after annealing remained in the range of 0.75 ± 0.05 µm (Figure 3c,e).

The most common chemical bonds in amorphous and nanocrystalline carbon films are sp^3^ and sp^2^ hybridizations. In the sp^3^ configuration, a carbon atom forms four sp^3^ orbitals making a strong *σ* bond to the adjacent atom. In the sp^2^ configuration, a carbon atom forms three sp^2^ orbitals forming three *σ* bonds and the remaining *p* orbital forms a *π* bond. The *π* orbital geometrically lies normal to the *σ* bond plane and is the weaker bond, and therefore it is closer to the Fermi level. The three *σ* bonds and *π* bond usually constitute a ring plane in sp^2^ clusters. An XPS analysis is a very sensitive method that can be used to identify and determine the concentration of elements within the escape depth of the photoelectrons in the near surface region. Because it can reveal the binding energy of the carbon atoms and discern the hybrid sp^3^ and sp^2^ bonds, it is a very powerful method for evaluating the structure of carbon films without causing excessive damage to the materials [38]. The XPS analysis evaluates the emitted electrons from the surface, and from the emitted electrons, we are able to characterize a binding energy which is typical for each element.

**Figure 4:**
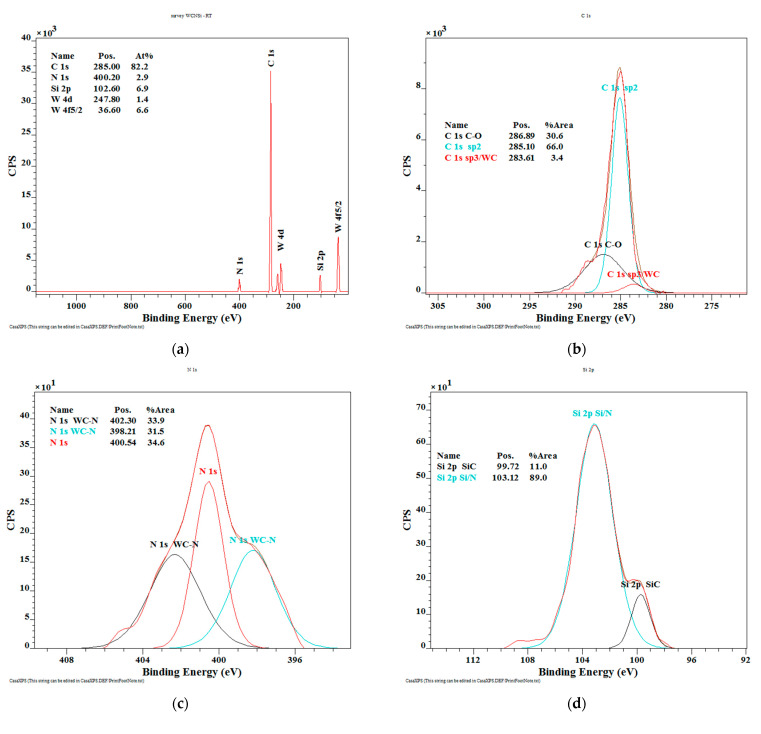

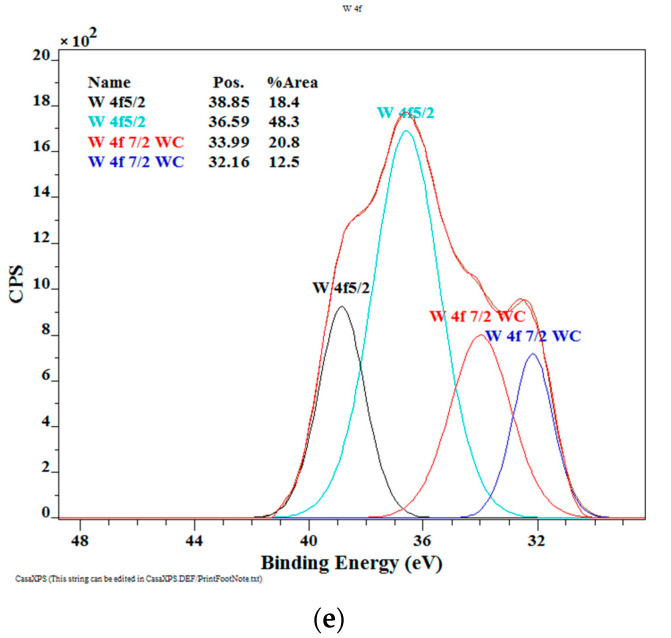
The XPS spectra of the deposited WC/C coatings: (**a**) Chemical composition (at.%); (**b**–**e**) chemical bonds of selected elements.

The XPS spectrum of the WC/C deposited unannealed coating (Figure 4a) shows the presence of C 1s 82.2 at.%, N 1s 2.9 at.%, Si 2p 2.9 at.%, and tungsten 8.0 at.%.

In addition, the obtained results (Figure 4b) show the presence of hard WC and diamond phase (3.4 at.%), graphite phase of C (66.0 at.%), and phase of C-O (30.6 at.%). The high-resolution spectrum of N 1s nitrogen (Figure 4c) shows the presence of hard WC-N (65.4 at.%) and nitrogen oxide (34.6 at.%). The spectrum of silicon Si 2p contains two peaks (Figure 4d), and peak with a value of 99.72 eV (11.0 at.%), which is characteristic for the SiC phase and confirms the binding of silicon in a thin layer. A peak of 103.12 eV (89.0 at.%) is characteristic for the silicon nitride phase. The high-resolution spectrum of W 4f tungsten is divided into four peaks (Figure 4e), i.e., peaks with values of 38.85 eV (18.4 at.%) and 36.59 eV (48.4 at.%) are characteristic for nitrogen-bound tungsten. In addition, the tungsten carbide compound is confirmed by peaks of 33.99 eV and 32.16 eV (20.8 at.% and 12.5 at.%). These results are in good agreement with results for C deconvolution [39], N convolution [40], and W convolution [41].

This is beneficial for the creation of chemical compounds, such as WSi_2_, SiC, SiO_2_, and Si_3_N_4_. These phases in the obtained structure during deposition and after annealing caused an increase in the refractoriness properties of the WC/C coating. Changes in the structure of the coating related to chemical reactions took place during the process of annealing at 500 °C without the protective atmosphere.

**Figure 5:**
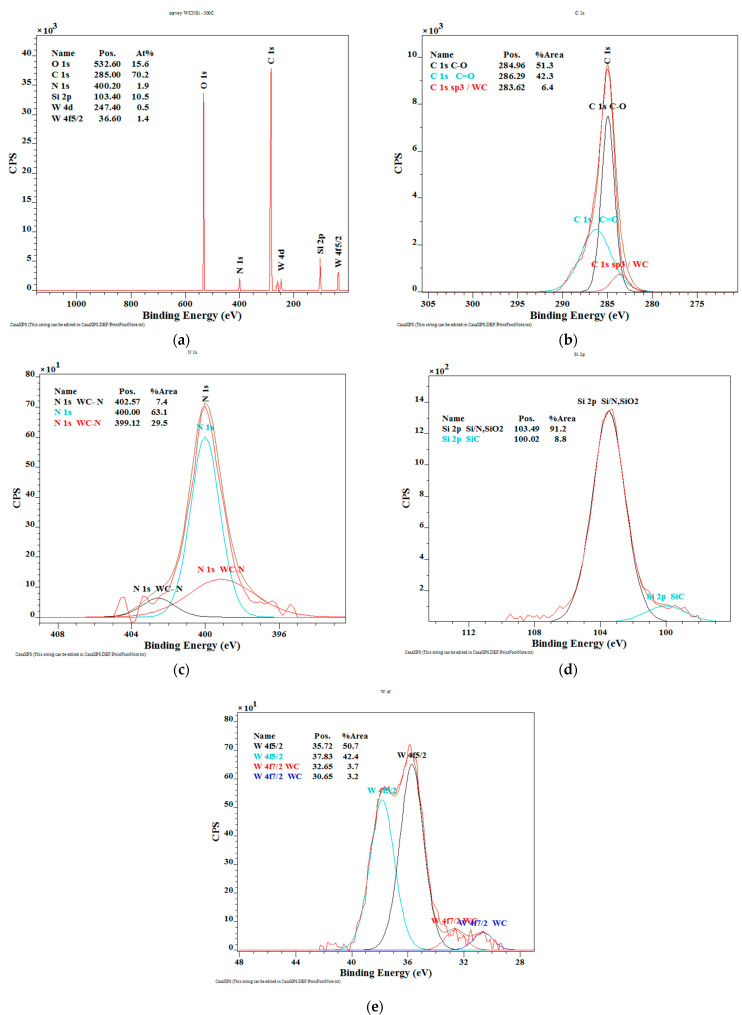
The XPS surface results of the WC/C coating annealed at 500 °C (at.%): (**a**) chemical composition; (**b**) chemical bonds C; (**c**) chemical bonds N; (**d**) chemical bonds Si; (**e**) chemical bonds W.

As shown in Figure 5a, the surface spectra of the WC/C coatings after annealing at 500 °C show the presence of oxygen, carbon, nitrogen, silicon, and tungsten, i.e., O 1s 15.6 at.%, C 70.2 at.%, N 1s 1.9 at.%, Si 2p 10.5 at.%, and W 1.9 at.%, respectively.

The high-resolution spectrum of C 1s carbon (Figure 5b) can be decomposed into three peaks, a peak of 286.29 eV (42.3 at.%), which is characteristic for the carbon in the C = O bond; the carbon bonded with oxygen from the air, a peak of 284.96 eV is attributed to the C-O bond (51.3 at.%); and the peak at 283.61 eV is typical for sp^3^ hybridization, which is characteristic for the diamond phase and the tungsten carbide compound (6.4 at.%). The presence of nitrogen (Figure 5c) on the surface of the coating confirms the high-resolution N 1s spectrum. From the given spectrum, we can confirm the bonding of the nitrogen, carbon, and tungsten in a thin layer. The value of 400.00 eV is attributed with nitrogen oxide. The high-resolution spectrum of silicon Si 2p (Figure 5d) contains two peaks. A line with a value of 99.72 eV (8.8 at.%) is characteristic for the SiC compound, which confirms the binding of silicon in a thin layer. A peak of 103.12 eV (91.2 at.%) is characteristic for silicon oxide.

The high-resolution spectrum of tungsten W 4f (Figure 5e) is divided into four peaks, i.e., values of 3572 eV (50.7 at.%) and 3783 eV (42.4 at.%) are characteristic for oxygen-bound tungsten. The tungsten carbide compound is confirmed by a peak at the value of 32.64 eV (3.7 at.%) and at the value of 30.65 eV (3.2 at.%).

**Figure 6:**
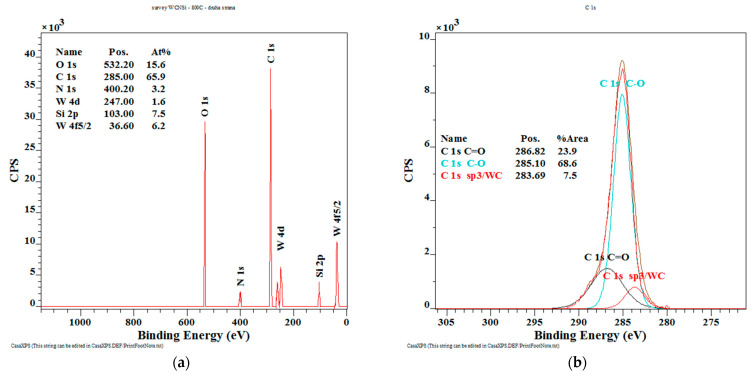

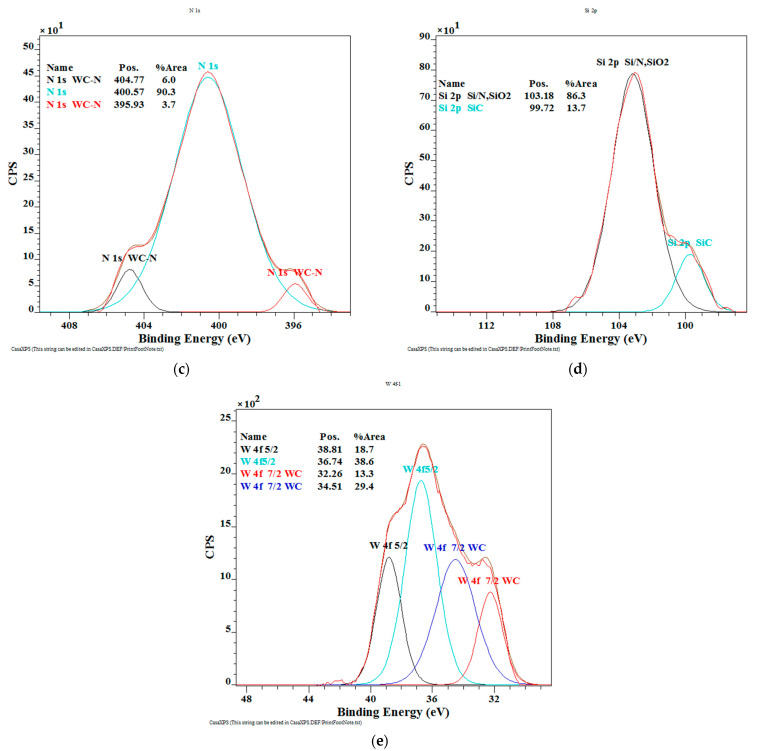
The XPS results from the surface of the WC/C annealed coating at 800 °C (at.%): (**a**) Chemical composition; (**b**) chemical bonds C; (**c**) chemical bonds N; (**d**) chemical bonds Si; (**e**) chemical bonds W.

As is shown in Figure 6a (after annealing at 800 °C), the surface spectra of the WC/C coatings show the presence of O 1s 15.6 at.%, C 1s 65.9 at.%, N 1s 3.2 at.%, Si 2p 7.5 at.%, and tungsten 7.8 at.%.

The high-resolution spectrum of C 1s carbon (Figure 6b) can be decomposed into three peaks, a peak of 286.82 eV (23.9 at.%), which is characteristic for the carbon in the C = O bond; the carbon bonded with oxygen from the air, a peak of 285.1 eV-the given peak is attributed to the C-O bond (68.6 at.%); and the peak at 283.69 eV is typical for sp^3^ hybridization, which is characteristic for the diamond phase and the tungsten carbide compound (7.5 at.%).

The presence of nitrogen in the tested sample confirms the high-resolution N 1s spectrum (Figure 6c), the peak at the value of 400.57 eV (90.3 at.%) belongs to nitrogen oxide. The high-resolution spectrum of silicon Si 2p (Figure 6d) contains two peaks, with a value of 99.72 eV (13.7 at.%) characteristic for the SiC phase. The peak of 103.18 eV (86.3 at.%) is characteristic for silicon oxide in the obtained structure.

The high-resolution spectrum of W 4f tungsten (Figure 6e) is divided into four peaks, with a value of 38.81 eV (18.7 at.%) and with a value of 36.74 eV (38.6 at.%), which are characteristic for oxygen-bound tungsten. In addition, the tungsten carbide compound is confirmed by peaks of 32.26 eV and 34.51 eV (13.3 at.% and 29.4 at.%).

### 3.2. Hardness and Young’s Modulus

**Figure 7:**
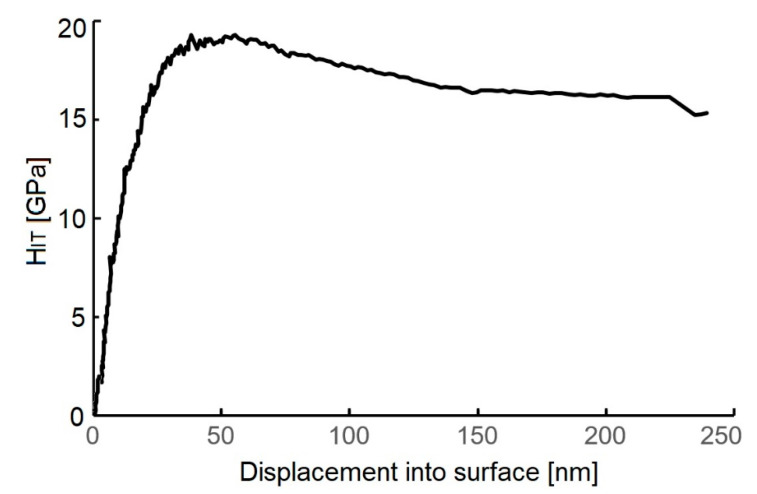
Depth profile of the H_IT_ values of the tested WC/C coating.

Figure 7 shows a depth profile of the indentation hardness course of the tested WC/C coating deposited using the N_2_-SiH_4_-Ar gas mixture.

**Figure 8:**
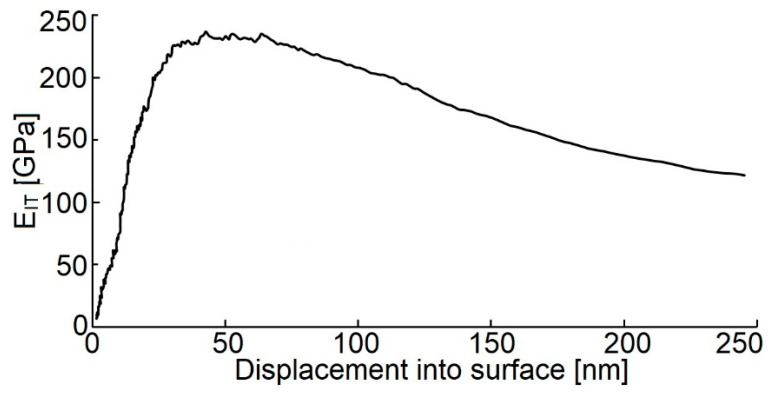
Depth profile of the E_IT_ values of the tested WC/C coating.

The maximum H_IT_ value of the deposited coating is 18.7 ± 4.3 GPa as compared with that obtained by other studies, namely [8] (18.0 ± 3.1 GPa) and [17] (22.1 ± 2.5 GPa), where thin layers were obtained using the DCMS and RFMS techniques, respectively. Simultaneously, the hardness is 30% lower as compared with the hardness of the WC/C coatings deposited using the PE CVD method with and without adding Ar in a gas mixture [27]. Young´s modulus of the tested samples is 220 ± 17 GPa (Figure 8).

**Figure 9:**
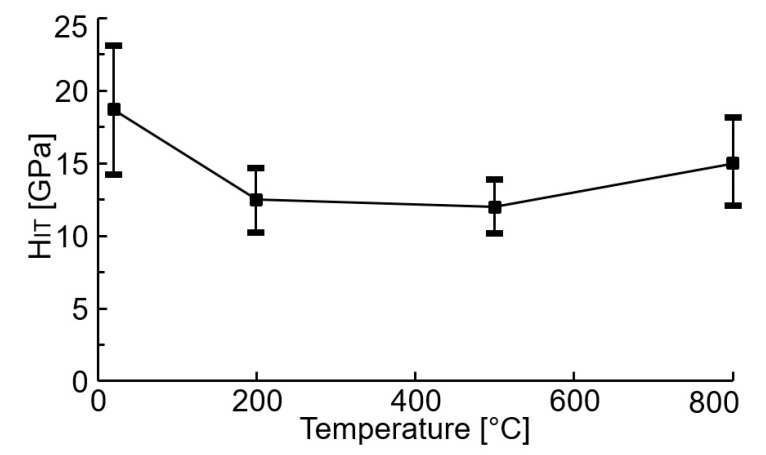
The H_IT_ values vs. the annealing temperature of the tested WC/C coatings.

After annealing at 200 °C, the H_IT_ decreased to 12.5 ± 2.3 GPa (Figure 9). The hardness of the coating did not change after annealing the coated substrate at 500 °C. A slight increase in hardness to 15.0 ± 2.7 GPa can be spotted after annealing the coated substrate at 800 °C, which is more than in the case of WC/C coating deposited using the PE CVD method with added Ar (12.0 ± 0.8 GPa) and significantly more than in the case of added N_2_ gas (3.0 ± 0.2 GPa) [27].

The mentioned difference could be attributed to the presence of WC, WN, W_2_N, SiC, and SiN which have been made in the process of deposition of the coating (Figure 4b–e) or during annealing, especially at a temperature of 800 °C (Figure 6b–e). It can also be stated that the mentioned differences correspond with the degree of distraction of the coating (as compared with the results by [27]) after annealing at the mentioned temperature. As compared with the results by [27], the least disrupted coating was the one which was deposited in the N_2_-SiH_4_-Ar atmosphere (Figure 3f).

### 3.3. Coefficient of Friction

**Figure 10:**
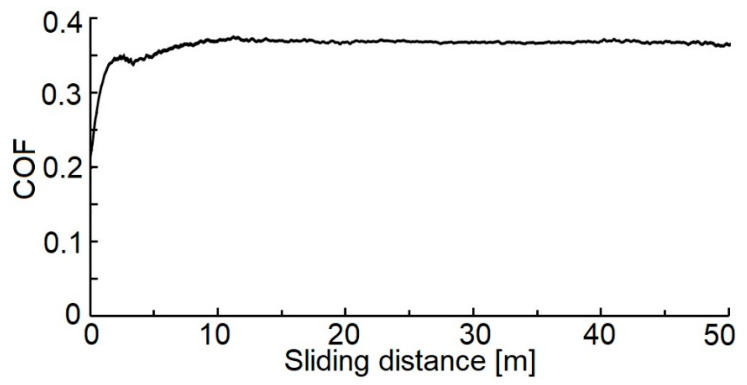
The COF values vs. the sliding distance of the tested WC/C coating.

Measures of the COF (0.35 ± 0.02, Figure 10) agree with other study results [8], where the achieved values of the COF of the WC/C coatings deposited with added N_2_ and Ar gas are equal to 0.58 ± 0.03 and. 0.77 ± 0.03, respectively.

If we compare the measured COF values of and the WC/C coatings deposited with the N_2_-SiH_4_ gas mixture using the DCMS (0.28 ± 0.03) [8] and RFMS (0.23 ± 0.02) [17] methods, it can be said that our measured value of COF increased by ca. 30%.

**Figure 11:**
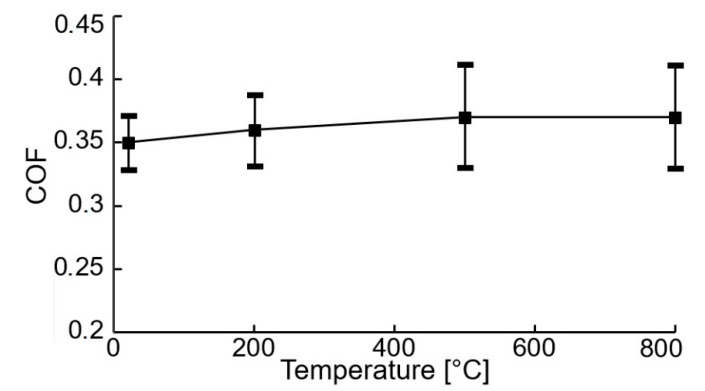
The COF values vs. the annealing temperature of the tested WC/C coatings.

In contrast, after the applied annealing process at a temperature of 200 °C, the COF value increased slightly to 0.36 ± 0.03 (Figure 11).

After annealing at 500 °C and 800 °C, the value of the COF was equal to 0.37 ± 0.40. This value increased by ca. 30% as compared with other study results (0.27 ± 0.02 [8] and 0.26 ± 0.01) [17] (annealing at a temperature of 500 °C) and by ca. 35% as compared with (0.22 ± 0.01) [17] (annealing at a temperature of 800 °C). It can be stated that the evaluated WC/C coatings deposited with the N_2_-SiH_4_ gas mixture have shown only an insignificant increase in the COF values after annealing at temperatures of 200, 500 and 800 °C (Figure 11).

The presented differences in the COF values (for the WC/C coating that has not been annealed) can be attributed to the amount of C in the graphite phase, which acts as a dry lubricant and causes a decrease in the COF value. In addition, regarding high COF values, C is present in harder particles, such as WC and WC_1-X_, WN and W_2_N, and even SiC, which in small amounts can be created during the coating deposition and, especially, during the annealing process at high temperatures (in our case at 800 °C). The presence of these harder particles in the WC/C coating causes an increase in the H_IT_ value. If a significantly softer counterpart is used, relative to the evaluated surface of the WC/C coating, the presence of harder particles in the WC/C coatings can cause an increase in the COF, which is attributed to an increase in contact surface during the tribological test.

## 4. Conclusions

According to the results of the tested samples of deposited WC/C coatings (unannealed and annealed at various temperatures), it can be stated that:

i. The obtained WC/C coatings using the PE CVD method are characterized by the following measurements of evaluated properties: H_IT_ = 18.7 ± 4.3 GPa, E_IT_ = 220 ± 17 GPa, and COF = 0.35 ± 0.02. In addition, higher values of hardness can be obtained by optimizing the technological process parameters.
ii. On the one hand, the annealing process causes a significant decrease in the *H_IT_* value, starting at 200 °C. On the other hand, after annealing at 500 and 800 °C, the hardness increases only insignificantly as compared with hardness after annealing at 200 °C.
iii. After annealing without a protective atmosphere at 500 °C, a slight coarsening of the granulate was spotted on the surface of the WC/C coating, creating bulgy particles with a diameter up to ca. 200 nm.
iv. The annealing process at 800 °C caused the creation of empty spaces in the coating with a diameter up to ca. 50 nm (less often up to ca. 400 nm), which were periodically located all over the surface of the coating. The oxidation process, which was partially accompanied by swelling, could have caused such an occurrence. This mechanism did not appear in the whole volume of the coating, but only in small regions.
v. The N_2_-SiH_4_-Ar gas mixture used during the deposition process of the WC/C coatings protects against significant degradation up to a temperature of 800 °C.

## Figures and Tables

**Table 1:** The technological parameters of the WC/C coating deposition using the PE CVD method using hexacarbonyl of W as the precursor and the selected mechanical properties of the obtained coating before annealing.

Type of Coating	Gas Mixture	Total Pressure (Pa)	Gas Pressure (Pa)	H_IT_ (GPa)	E_IT_ (GPa)	COF (-)
WC	N_2_-SiH_4_	4.0	2.0	18.7 ± 4.3	220 ± 17	0.35

## Data Availability

Data sharing is not applicable to this article.
